# Prevalence of lingual sensory impairment following bilateral sagittal split osteotomy: a systematic review and meta-analysis

**DOI:** 10.1007/s10006-024-01247-w

**Published:** 2024-04-16

**Authors:** Evangelos Kostares, Michael Kostares, Georgia Kostare, Maria Kantzanou

**Affiliations:** 1 Department of Microbiology, Medical School, National and Kapodistrian University of Athens, 115 27 Athens, Greece; 2 National and Kapodistrian University of Athens Faculty of Medicine: Ethniko kai Kapodistriako Panepistemio Athenon Iatrike Schole, 115 27 Athens, Greece

**Keywords:** Lingual nerve injury, Lingual sensory impairment, Bilateral sagittal split osteotomy, Mandibular osteotomies, Prevalence, Meta-analysis

## Abstract

**Purpose:**

Our study aims to estimate the prevalence of lingual nerve injury following bilateral sagittal split osteotomy (BSSO).

**Methods:**

Two reviewers independently conducted a systematic literature search in the Medline and Scopus databases. The pooled prevalence with 95% confidence intervals (CI) was estimated, and quality assessment, outlier analysis, and influential analysis were performed.

**Results:**

In total, eleven eligible studies comprising a total of 1,882 participants were included in this meta-analysis. One study was identified as critically influential. The overall prevalence of lingual sensory impairment was estimated to be as high as 0.1% (95% CI 0.0%-0.6%) with moderate heterogeneity observed between studies.

**Conclusion:**

It is important for healthcare professionals to be aware of this issue, despite the relatively low rate of lingual nerve deficit after BSSO. Additional research will provide a more comprehensive understanding of the underlying factors contributing to lingual nerve injury, leading to improved preventive measures and treatment strategies. Furthermore, insights gained from future studies will enable healthcare professionals to inform patients about the potential complications and manage their expectations before undergoing BSSO.

**Supplementary Information:**

The online version contains supplementary material available at 10.1007/s10006-024-01247-w.

## Introduction

Orthognathic surgery is a surgical procedure aimed at rectifying facial skeletal components and restoring the normal anatomical and functional connections in individuals with dentofacial skeletal abnormalities. These abnormalities derive from either the dento-alveolar complex, the skeletal base, or both, and can manifest as horizontal mandibular excess, deficiency, and/or asymmetry. They manifest in three distinct manners: antero-posterior, transverse, and vertical orientations. Before delving into the classification of jaw deformities, it is imperative to meticulously evaluate the interrelationship between the lower jaw and the rest of the face. Multiple objective parameters are employed to assess the deformity, with clinical evaluation holding paramount importance [1–3]. A significant aspect of orthognathic surgery involves the utilization of bilateral sagittal split osteotomy (BSSO), which is the prevailing procedure for jaw surgery, whether performed independently or in conjunction with upper jaw surgery. The indications for a bilateral sagittal split encompass cases of horizontal mandibular excess, deficiency, and/or asymmetry. This particular technique is widely employed for mandibular advancement, serving as the primary method, and can also be utilized for modest to moderate mandibular setback procedures [3]. Numerous complications are associated with BSSO such as the risk of improper split, potential injury to the neurovascular bundle, temporomandibular joint (TMJ) issues, excessive bleeding, and the possibility of relapse [1, 32]. The lingual nerve, a derivative of the mandibular division (V3) of the trigeminal nerve (CN V), supplies sensory information to the lingual gingiva and the anterior two-thirds of the tongue. It is important to acknowledge that the chorda tympani nerve, which is responsible for gustatory perception in the anterior two-thirds of the tongue, converges with the lingual nerve at the level of the lower border of the lateral pterygoid muscle. It is imperative to recognize that damage to the lingual nerve can potentially harm the chorda tympani nerve, leading to changes in taste and sensory perception on the affected side. Individuals affected by such injuries commonly encounter significant discomfort during basic activities like chewing, eating, and speaking. The specific character and severity of altered sensations can vary significantly among individuals, encompassing a variety of symptoms such as paresthesia (unusual sensations like pins and needles), hypesthesia (reduced or complete loss of sensation), and dysesthesia (abnormal sensations such as pain) [4–6]. In the context of orthognathic surgery, a crucial knowledge gap arises regarding the prevalence of lingual nerve injury following bilateral sagittal split osteotomy (BSSO). This gap is apparent due to the substantial heterogeneity observed across scientific publications [7–9], underscoring the necessity for a more precise and comprehensive understanding of lingual nerve damage in the aftermath of BSSO procedures. Consequently, the objective of the present investigation is to provide a more accurate assessment of the occurrence of lingual nerve damage following BSSO, through a meta-analysis of the existing data found in the scientific literature.

## Methods

### Search strategy

**Fig. 1:**
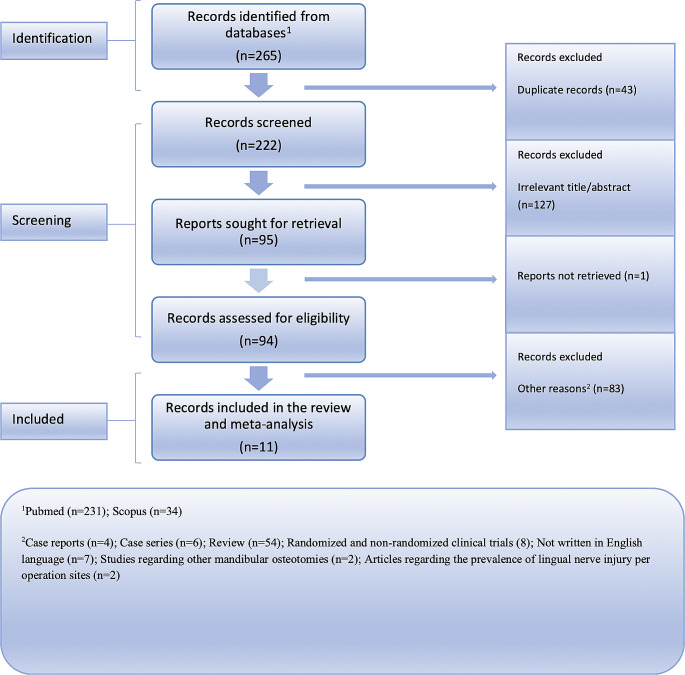
Flow chart depicting the systematic search results from the relevant studies’ identification and selection

The Medline (PubMed search engine) and Scopus database were comprehensively searched following the Preferred Reporting Items for Systematic Reviews and Meta-Analysis (PRISMA) guidelines [10] to ensure a rigorous approach (Fig. 1). The PRISMA checklist, available in Supplementary materials (Supplementary Table 1), was utilized to facilitate the systematic review process. We have collected articles that were published up until May 1st, 2023. The literature search was independently performed by two reviewers using a combination of the following keywords: “*lingual nerve injury*”, “*lingual sensory impairment*”, “*lingual nerve damage*”, “*bilateral sagittal split osteotomy*”, “*BSSO*”, “*ramus osteotomy*”, “*mandibular osteotomies*”, “*prevalence*”, “*incidence*”, “*rate*”. In conjunction with the primary search, a thorough examination of the reference lists from the identified studies was conducted to identify any additional articles that may have been overlooked. The collected studies were meticulously organized and stored using the Zotero reference management software (version 6.0.18) [11]. We ensured the credibility of our dataset by diligently removing any duplicate references. Following the initial search, two independent investigators thoroughly examined the remaining articles. The study selection process consisted of two distinct stages. Initially, we meticulously reviewed the titles and abstracts of the articles, eliminating those that did not meet our predetermined criteria for inclusion. In the second stage, we obtained the full texts of the remaining articles and conducted a comprehensive evaluation. Differences in study selection were resolved through iterative discussions and consensus-building among the team members. In instances where there were differing opinions or interpretations, the team engaged in thorough deliberations to reach a shared understanding and agreement on whether a particular study met the predetermined inclusion criteria. This collaborative approach ensured a transparent and unified decision-making process throughout the study selection phase.

### Criteria for study selection and data extraction

In our selection process, we focused on observational studies (cross-sectional, cohort) specifically examining the prevalence rates of lingual nerve injury following BSSO procedures. We did not impose any restrictions on publication dates. Case reports, case series with less than five participants, review articles, randomized clinical trials, animals studies, letters to the editor, books, expert opinion, conference abstracts, studies with no full-text available, studies not written in English, studies regarding other mandibular osteotomies [12], articles regarding the prevalence of lingual nerve injury per operation sites [13] and articles containing data derived from surveillance databases were excluded. In articles with overlapping populations, the most recent or most complete publication was considered eligible. The following variables were obtained from each study: the first author’s name, year of publication, study design, continent of origin, study period, total patients, proportion of males, mean age, patients with postoperative lingual nerve injuries and diagnostic procedure performed.

### Quality assessment

To evaluate the quality of the studies included, two investigators independently assessed them using the National Heart, Lung, and Blood Institute (NHLBI) Quality Assessment tool for Observational Cohort and Cross-Sectional Studies. The evaluation process entailed a thorough examination of each study to identify any methodological or survey implementation weaknesses that could impact internal validity. During the assessment, the investigators considered fourteen specific questions to gauge the quality of each study. They were provided with response options such as “yes,” “no,” “cannot determine” (e.g., in instances where the data presented uncertainties or contradictions), “not reported” (e.g., in cases where data were not reported or were incomplete), or “not applicable” (e.g., when a question did not pertain to the specific type of study under evaluation). By evaluating these questions, the investigators categorized the risk of bias for each study as either “low,” “moderate,” or “high,” enabling an overall assessment of the study’s quality [14]. By conducting this rigorous quality appraisal, our aim was to ensure that only studies demonstrating a moderate or high level of internal validity were included in our analysis.

### Statistical analysis

Statistical analysis was carried out using RStudio (version: 2022.12.0 + 353) software (RStudio Team (2022) [15]. The meta-analysis was conducted through metafor package [16]. The DerSimonian and Laird random-effects model was used to estimate the pooled prevalence and its respective 95% confidence intervals (CI) (a random-effects model assumes each study estimates a different underlying true effect). Freeman-Tukey double arcsine transformation was performed [17]. Heterogeneity presence between studies was evaluated through visual inspection of the forest plot and by using the Cochran’s Q statistic and its respective *p* value. The Higgins I^2^ statistic and its respective 95% CI were used for quantifying the magnitude of true heterogeneity in effect sizes. An I^2^ value of 0-40%, 30-60%%, 50-90% and 75-100% indicated not important, moderate, substantial and considerable heterogeneity, respectively [18]. To determine if the potential outlying effect sizes were also influential, screening for externally studentized residuals with z-values larger than two in absolute value and leave-one-out diagnostics were performed [19]. Due to paucity of data regarding categorical and continuous variables, such as proportion of males, mean age and duration of surgery subgroup and meta-regression analysis were not performed [20]. Unless otherwise stipulated, the statistical significance was established at *p* = 0.05 (two-tailed). Tests to evaluate publication bias, such as Egger’s test [21], Begg’s test [22] and funnel plots, were developed in the context of comparative data. They assume studies with positive results are more frequently published than studies with negative results, however in a meta-analysis of proportions there is no clear definition or consensus about what a positive result is [23]. Therefore, publication bias in this current meta-analysis was assessed qualitatively.

## Results

### Results and characteristics of the included studies

In total, eleven studies (comprising a sum of 1,882 participants) were finally included in this analysis. The descriptive characteristics of them are reported in Table 1. All articles were published from 1995 to 2022 (conducted from 1980 to 2020). Two of them were of retrospective cohort design and the remaining ones of cross-sectional. Most of the studies were carried out in Europe (The Netherlands, Sweden, Finland, Italy, Belgium, Germany), followed by America (USA) and Asia (Japan). The average percentage of males was 40.8% and the mean age of participants ranged from 19.9 years to 35 years (median: 26.3years). As per the quality assessment, all of them were estimated as moderate quality.

**Table 1:** Descriptive characteristics of the included studies

First author	Year of publication	Study design	Continent of origin	Country	Study period	Total patients	Proportion of males (%)	Mean age (years)	Lingual nerve injury	Diagnostic procedure performed	Quality assessment
Bowman J.P.B [24]	1995	cross-sectional	Europe	The Netherlands	NA	700	NA	NA	4	NA	Moderate
August M [25]	1998	cross-sectional	America	USA	1985–1995	85	31.8	30.9	2	Subjective	Moderate
Jacks S.C [26]	1998	cross-sectional	America	USA	1980–1993	134	18.7	29.7	8	Subjective	Moderate
Al-Bishri A [27]	2004	cross-sectional	Europe	Sweden	1995–2000	93	40.9	35	1	Subjective	Moderate
Kallela I [28]	2005	cross-sectional	Europe	Finland	NA	40	27.5	29	0	Subjective	Moderate
Matsushita Y [29]	2015	retrospective cohort	Asia	Japan	2006–2012	75	46.7	25.8	1	Objective	Moderate
Posnick J.C [30]	2016	retrospective cohort	America	USA	2004–2013	262	49	25	1	Subjective, Objective	Moderate
D’Agostino A [7]	2019	cross-sectional	Europe	Italy	2013–2015	52	58	26.3	0	Subjective	Moderate
da Costa Senior O [8]	2020	cross sectional	Europe	Belgium	2013–2016	376	35	26	0	Objective	Moderate
Thiem D.G.E [9]	2021	cross-sectional	Europe	Germany	2010–2016	45	NA	NA	0	Objective	Moderate
Sobol D.L [31]	2022	cross-sectional	America	USA	2017–2020	20	60	19.9	0	Objective	Moderate

NA: not applicable

### Prevalence of lingual nerve injury following BSSO

**Fig. 2:**
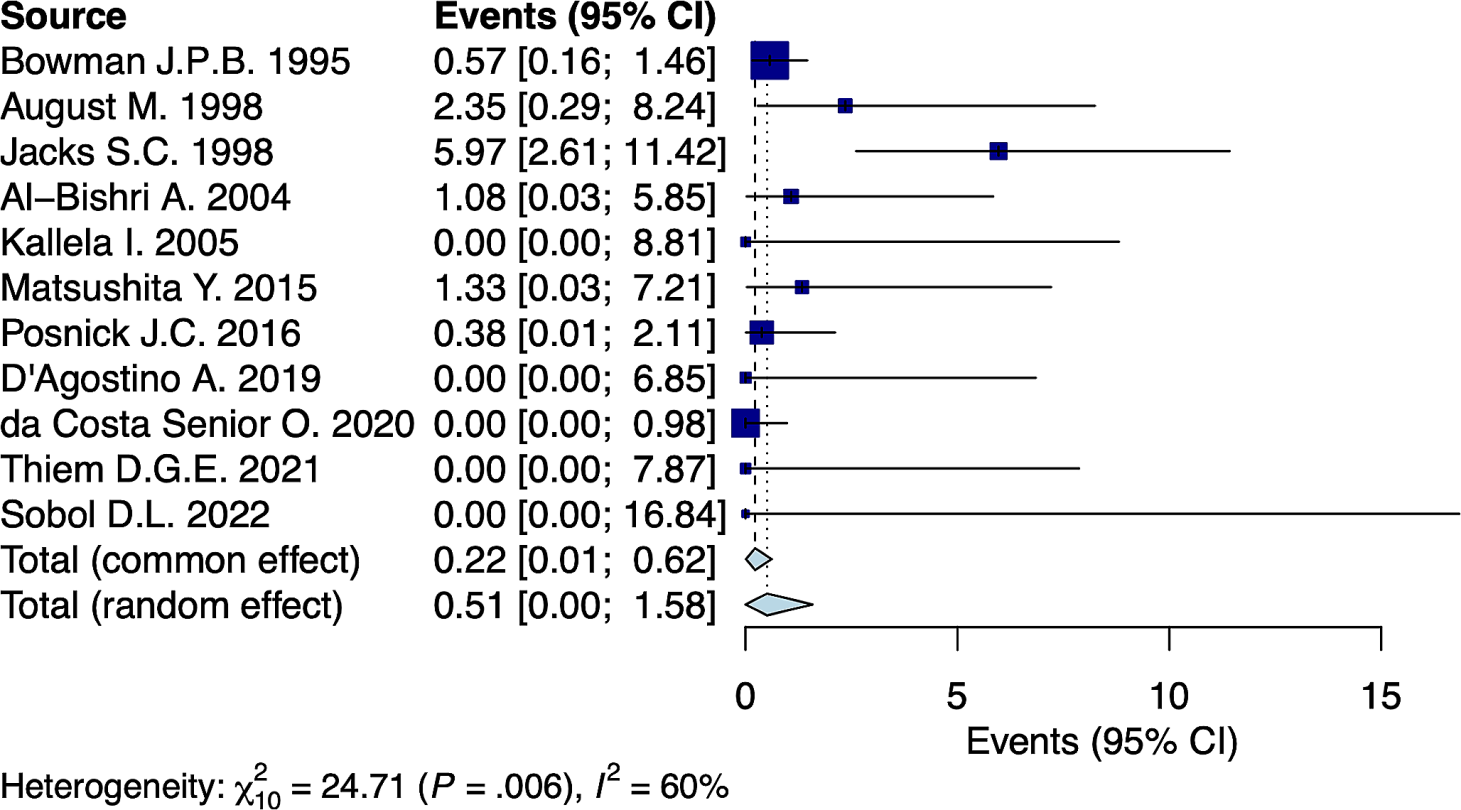
Forest plot evaluating the calculated prevalence of lingual nerve injury after BSSO using random-effects model

A random-effects model analysis yielded an initial overall lingual nerve injury prevalence following BSSO of 0.5% (95%CI 0.0-1.6%) with considerable between studies heterogeneity I^2^ = 60% (95%CI 15-86%, *p* = 0.006) (Fig. 2). The influence diagnostics and the forest plot illustrating the results of the leave-one-out analysis is presented in Supplementary materials (Supplementary Fig. 1, Supplementary Fig. 2). As per them, the study conducted from Jacks S.C., et al. [26] identified as influential. After the exclusion of the aforementioned study the estimated prevalence was calculated at 0.1% (95%CI 0.0-0.6%) with moderate between studies remaining heterogeneity I^2^ = 1% (95%CI 0-58%) (*p* = 0.43).

## Discussion

The prevalence of lingual nerve injury after BSSO has garnered considerable attention from clinicians and researchers alike. Numerous studies have been conducted to investigate this complication, seeking to determine its incidence and potential risk factors. According to the results of our study, the prevalence of lingual nerve impairment following BSSO is estimated at 0.1% (95%CI 0.0-0.6%) with moderate between studies remaining heterogeneity. Our attempts to perform subgroup and meta-regression analyses were impeded by the insufficiency of available data pertaining to potential risk factors. The remaining heterogeneity could be ascribed to the diagnostic procedure carried out to assess lingual nerve injury. In the majority of the studies, exclusively subjective methods, such as questionnaires, were employed. Evaluation of lingual nerve injury can be approached through both subjective and objective methods. Subjective methods involve relying on the patient’s reported symptoms and experiences, whereas objective methods rely on clinical assessments and diagnostic tests. The objectives test can be categorized into two groups: mechanoreceptive and nociceptive. The evaluation of mechanoceptive testing involves non-painful stimuli, such as static light touch, brush directional stroke, and two-point discrimination. On the other hand, nociceptive testing concentrates on assessing responses to pinpricks and thermal discrimination [6, 32]. Moreover, it is worth emphasizing that significant heterogeneity is expected in prevalence and incidence estimates due to the type of this study (differences in the time and place where included studies were conducted). Therefore, high I^2^ in the context of proportional meta-analysis does not necessarily mean that data is inconsistent [23, 33].

To the best of our knowledge, there is only a sole meta-analysis to date related to this issue in the scientific literature, Shawky M., et al. [34], using data from three studies estimate the prevalence of lingual nerve damage after BSSO at 0.7% with substantial heterogeneity I^2^ = 66.7% (*p* = 0.365) between studies. Our estimation based on ten studies is lower 0.1% (95%CI 0.0-1.6%). Potential reasons for this discrepancy could be the larger number of studies used, different inclusion/exclusion criteria, quality assessment performed and the transformation of the data used in order to calculate the prevalence. Regarding the impairment of the lingual nerve in other surgical procedures, in a recent meta-analysis conducted by Lee J. et al. [35], the prevalence of lingual nerve injury following mandibular third molar extraction using various surgical approaches in the oral cavity was determined. The study reported that the buccal approach without lingual flap retraction had a prevalence of 0.18% for lingual nerve injury, whereas the buccal approach with lingual flap retraction showed a lower prevalence of 0.07%. Additionally, the lingual split technique exhibited a prevalence of 0.28% for lingual nerve injury.

Various therapeutic approaches, including nonoperative and surgical interventions, can be utilized following damage to the lingual nerve. Nonoperative treatments are typically regarded as the primary method of addressing long-standing injuries or pain. The primary objectives of nonoperative treatment involve pain reduction, addiction prevention, avoidance of surgical procedures with limited success rates, and enhancement of the patient’s quality of life. Nonoperative care encompasses both behavioral and pharmacologic modalities. The decision to pursue microsurgical treatment is determined on an individual basis, considering the specific presentation and clinical progression of each patient. The selection of treatment for injured lingual nerves (LNs) depends on factors such as the nature of the injury, the timing of the injury, neurosensory disturbances, and intraoperative findings. Optimal recovery of nerve function is achieved when nerve endings with gaps smaller than 10 mm are directly joined and sutured, while larger gaps necessitate nerve grafting. The optimal timing for microneurosurgical repair following an injury remains a topic of ongoing debate [4, 36]. As a result, healthcare practitioners should be knowledgeable about this matter, even though the occurrence of lingual nerve deficit after BSSO is relatively infrequent. Further investigation will enhance our understanding of the underlying elements that contribute to lingual nerve injury, thereby facilitating the improvement of preventive techniques and treatment approaches. By acquiring a clearer picture of its prevalence and related risk factors, we can establish guidelines that minimize the occurrence of lingual nerve impairment. Moreover, the findings from upcoming studies will empower healthcare professionals to educate patients about potential complications and effectively manage their expectations prior to undergoing BSSO. In conclusion, despite its limitations, our study has the potential to be utilized as a valuable prevalence index for everyday clinical practice and as a foundation for future research.

### Study’s strengths and limitations

The main strength of the current study was the comprehensive methodology applied for the literature search, study selection, inclusion/exclusion criteria, screening for eligibility, quality assessment and pooling analysis of prevalence data from eleven studies. However, the present study had several limitations. It should be noted that the unidentified heterogeneity remained moderate, therefore, the results should be interpreted with caution. The heterogenous outcomes across the included studies were expected due to the nature of this type of studies. The subjectivity of the lingual nerve injury diagnosis among patients and other potential risk factors might bias the prevalence of lingual nerve damage after BSSO. Due to limited data (less than ten studies for each covariate) regarding variables such as mean age, proportion of males, duration of surgery, surgeon level, these variables were excluded from this presented analysis. Moreover, the deliberates inclusion of only observational studies conducted in English introduces a reporting bias, excluding valuable research conducted in languages other than English. This decision, while made for practical reasons, may inadvertently overlook a significant body of literature, especially in regions with limited resources where research is often conducted in local languages. Consequently, the study’s findings may not comprehensively reflect the global landscape of relevant research, potentially limiting the generalizability and applicability of the results. Only studies from Europe, America, and Asia were finally included in our analysis. Furthermore, it is critical to acknowledge that the inherent risk of lingual nerve injury is not directly due to the sagittal osteotomy procedure itself, as the lingual nerve is anatomically positioned outside the bone’s path. Instead, the potential for nerve damage predominantly arises from procedural aspects such as the incision, flap elevation, and meticulous surgical handling. Lastly, our meta-analysis was not registered in PROSPERO, which may be a source of reporting bias. In light of these limitations, it is important to note again that the results should be interpreted cautiously due to the limited generalizability of the data and the potential underestimation or overestimation of the prevalence.

## Electronic supplementary material

Below is the link to the electronic supplementary material.

Supplementary Material 1

## Data Availability

Literature and Rstudio data are available from the corresponding author on reasonable request.
